# Sex Differences in In-hospital Mortality of Patients With Septic Shock: An Observational Study Based on Data Analysis From a Cover Sheet of Medical Records in Beijing

**DOI:** 10.3389/fmed.2021.733410

**Published:** 2021-10-11

**Authors:** Xiao Zhou, Na Zeng, Pei Liu, Zhuang Liu, Meili Duan

**Affiliations:** ^1^Department of Critical Care Medicine, Beijing Friendship Hospital, Capital Medical University, Beijing, China; ^2^Clinical Epidemiology and EBM Unit, National Clinical Research Center for Digestive Disease, Beijing Friendship Hospital, Capital Medical University, Beijing, China

**Keywords:** septic shock, sex, in-hospital mortality, risk factor, cover sheet of medical records

## Abstract

**Background:** The goal of our study was to evaluate the association of sex and in-hospital mortality in patients with septic shock in Beijing, China.

**Materials and Methods:** We analyzed 3,643 adult patients with septic shock from January 1, 2019, to Dec 31, 2019, in all secondary and tertiary hospitals in Beijing. Study data were retrospectively extracted from the Quality Control Center of Beijing Municipal Health Commission.

**Results:** There were 2,345 (64.37%) male and 1,298 (35.63%) female patients. Compared to male patients, female patients with septic shock had a higher in-hospital mortality rate (55.54 vs. 49.29%, *p* < 0.01). The median length of hospitalization stay for male patients was 22.71 days, while that for female patients was 19.72 days (*p* > 0.01). Male patients had a higher prevalence of pulmonary infection (68.8 vs. 31.2%, *p* < 0.01). The B values of sex in univariate and multivariate logistic regression were −0.251 and −0.312, respectively. Men had a lower likelihood of hospital mortality than women (OR = 0.732, 95% CI = 0.635–0.844, *p* = 0.000).

**Conclusions:** Female patients with septic shock had a higher risk of dying in the hospital than male patients.

## Introduction

Sex is increasingly recognized as a key factor in trauma (1), coronary heart disease (2), autoimmune disease (3), cancer, mental disorder (4) and other medical conditions. A number of studies suggest that a patient's gender may influence both the provision of care as well as outcomes. Critical care is not immune to such bias (5).

Sepsis is a life-threatening organ dysfunction caused by a dysregulated host response to infection (6). Septic shock is a complex inflammatory crisis associated with a high rate of mortality (7). Sepsis and septic shock are major health care problems, affecting millions of people around the world each year, resulting in the death of as many as one in four patients (and often more) (6). Recently, several studies have evaluated the effect of gender for patients with sepsis or septic shock. However, reports on the sex and mortality of sepsis/septic shock have shown conflicting results (8–11). The goal of this study was to evaluate the association of sex and in-hospital mortality in patients with septic shock in Beijing, China.

## Materials and Methods

### Study Population

**Figure 1:**
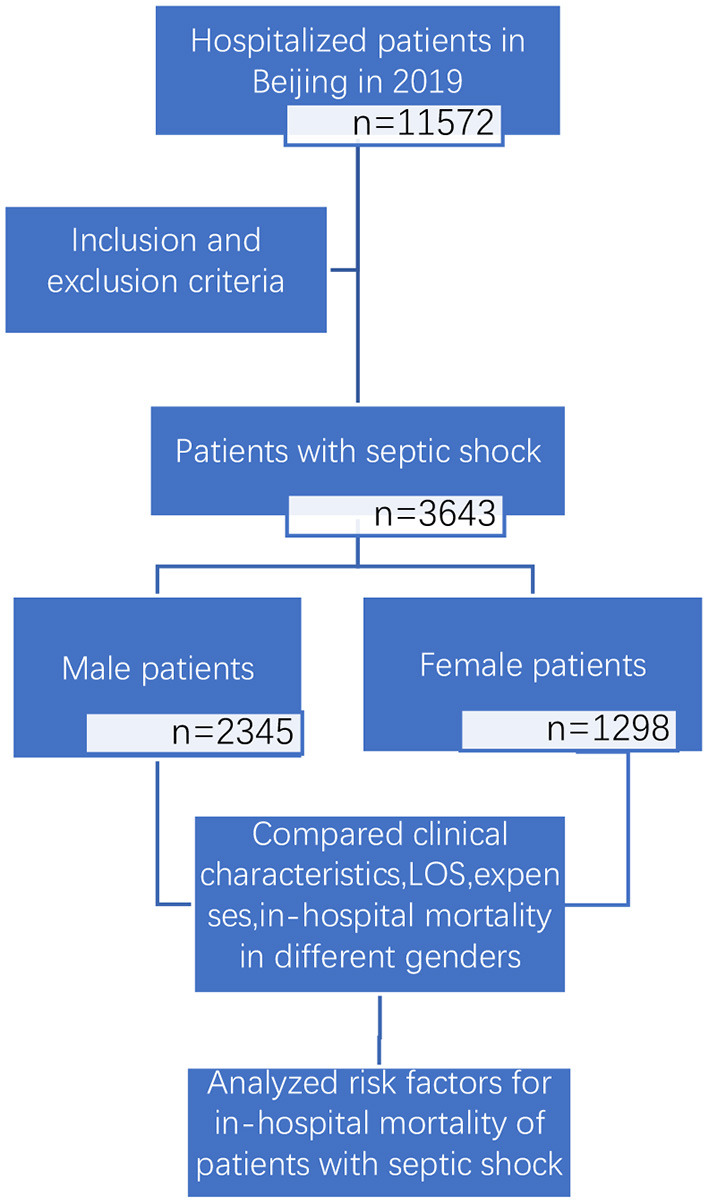
The study flow chart.

We conducted a retrospective and observational study (study flow chart shown in Figure 1). Based on the principal discharge diagnosis, patients with septic shock based on the sepsis-3.0 definition were enrolled by reviewing the inpatient lists from January 1, 2019, to Dec 31, 2019, in all secondary and tertiary hospitals in Beijing. The only exclusion criterion was an age <18 years old. This study was approved by the ethics committee of Beijing Friendship Hospital (No. 2021-P2-184-01) and granted a waiver of informed consent.

### Data Collection

Study data were retrospectively extracted from the Quality Control Center of Beijing Municipal Health Commission. Data elements were collected from the cover sheet of medical records, including patient' demographics, medical history, expenses, length of hospital stay, hospital level and diagnosis discharge form.

### Study Variables

Discharge forms included the following: recovered and discharged, discharged without recovery, referral, and death. We defined the first three conditions as “alive” and calculated the in-hospital mortality of patients with septic shock.

The race of patients was categorized as “Han” or “non-Han”. Hospital levels were categorized as “Tertiary hospitals” or “Secondary hospitals” which were determined officially.

The insurance of patients was categorized as “medical insurance” or “self-pay”. “Medical insurance” included Urban Employee Basic Medical Insurance and Urban Resident Basic Medical Insurance, New Rural Cooperative Medical Insurance or Business insurance.

Comorbidities included hypertension, diabetes mellitus, ischemic heart disease, chronic kidney disease, liver disease, chronic pulmonary disease and malignant tumors. Hypertension was defined as having a history of hypertension, receiving antihypertensive therapy, or having a systolic blood pressure ≥140 mmHg or diastolic blood pressure ≥90 mmHg on admission. Diabetes mellitus was defined as having a previous or new diagnosis of diabetes mellitus, receiving oral hypoglycemic drug therapy or insulin therapy, or having a fasting blood glucose level ≥7.0 mmol/L (126 mg/dL) or hemoglobin A1c level ≥6.5%. Ischemic heart disease included angina pectoris and myocardial infarction. Chronic kidney disease (CKD) was defined as abnormal kidney structure or function persisting for longer than 3 months. Liver disease included viral hepatitis and autoimmune, metabolic or alcohol-related liver disorders. Chronic pulmonary disease included chronic respiratory disease, cor pulmonale and pulmonary circulatory disease.

The site of infection included the pulmonary, skin, urinary tract, gastrointestinal tract, abdominal cavity and bloodstream.

The primary endpoint in the study was in-hospital mortality, which was defined as death during hospitalization. The hospital length of stay (LOS) and expenses were selected as the secondary outcomes.

### Statistical Methods

Categorical variables are presented as frequencies (n) and percentages (%). Continuous variables that conformed to a normal distribution are expressed as the mean ± standard deviation, and those that did not conform to a normal distribution are expressed as the median (interquartile range). An unpaired *t* test or Mann–Whitney *U* test was used to assess the statistical significance of differences between means or medians, where appropriate. The significance of differences for categorical variables was analyzed using the Chi-squared test. To evaluate the relationship between sex and in-hospital mortality, univariate and multivariate logistic regression analyses were performed. All statistical analyses were performed using SPSS version 25.0. Two-sided *P* < 0.01 were considered statistically significant.

## Results

### Patient Characteristics

Among 3,643 patients with septic shock who were included in this study, 2,345 (64.37%) were male, and 1,298 (35.63%) were female. The clinical characteristics of the study population are summarized in Table 1.

**Table 1:** Clinical characteristics of the septic shock patients at discharge.

	**Total (*n* = 3,643)**	**Men (*n* = 2,345)**	**Women (*n* = 1,298)**	**Test value**	***p*** **value**
**Age** [years, M(P25-P75)]	77.00 (62.00,85.00)	78.00 (62.00,85.00)	77.00 (63.00,84.00)	−1.257	0.209
**Marital status**				0.214	0.644
Unmarried (*n*, %)	228	150 (65.8%)	78 (34.2%)		
Married (*n*, %)	3,415	2,195 (64.3%)	1,220 (35.7%)		
**Race**				1.807	0.179
Han (*n*, %)	3,642	2,345 (64.4%)	1,297 (35.6%)		
Non-Han (*n*, %)	1	0	1 (100%)		
**Insurance**				3.283	0.070
Medical insurance (*n*, %)	3,304	2,142 (64.8%)	1,162 (35.2%)		
Self-pay (*n*, %)	339	203 (59.9%)	136 (40.1%)		
**Level of hospital**				0.958	0.328
Second hospital (*n*, %)	399	248 (62.2%)	151 (37.8%)		
Tertiary hospital (*n*, %)	3,244	2,097 (64.6%)	1,147 (35.4%)		
**Comorbidity**					
Hypertension (*n*, %)	1,803	1,174 (65.1%)	629 (34.9%)	0.861	0.354
Ischemic heart disease (*n*, %)	1,632	1,058 (64.8%)	574 (35.2%)	0.271	0.603
DM (*n*, %)	1,351	872 (64.5%)	479 (35.5%)	0.029	0.866
CKD (*n*, %)	1,201	809 (67.4%)	392 (32.6%)	6.986	0.008※
Malignant tumor (*n*, %)	707	498 (70.4%)	209 (29.6%)	14.086	0.000※
Liver disease (*n*, %)	1,347	917 (68.1%)	430 (31.9%)	12.807	0.000※
Chronic pulmonary disease (*n*, %)	180	120 (66.7%)	60 (33.3%)	0.435	0.509

※ *means that the differences is statistically significant (*P* value < 0.01)*.

The mean ages of male and female patients were 78.00 (62.00, 85.00) and 77.00 (63.00, 84.00) years, respectively. Men had a higher prevalence of malignant tumors (70.4 vs. 29.6%, *p* < 0.01), chronic kidney disease (67.4 vs. 32.6%, *p* < 0.01) and liver disease (68.1 vs. 31.9%, *p* < 0.01).

### Site of Infection in Different Genders

The main site of infection leading to septic shock was the pulmonary system. Men had a higher prevalence of pulmonary infection (68.8 vs. 31.2%, *p* < 0.01) (see Table 2).

**Table 2:** Site of infection in different genders.

**Site of infection**	**Total (*n* = 3,643)**	**Men (*n* = 2,345)**	**Women (*n* = 1,298)**	**Test value**	***p* value**
Pulmonary (*n*, %)	933	642 (68.8%)	291 (31.2%)	10.782	0.001※
Skin (*n*, %)	74	46 (62.2%)	28 (37.8%)	0.161	0.689
Urinary tract (*n*, %)	802	518 (64.6%)	284 (35.4%)	0.021	0.884
Gastrointestinal tract (*n*, %)	94	61 (64.9%)	33 (35.1%)	0.012	0.914
Abdominal cavity (*n*, %)	418	272 (65.1%)	146 (34.9%)	0.101	0.750
Blood stream (*n*, %)	74	46 (62.2%)	28 (37.8%)	0.161	0.689

※ *means that the differences is statistically significant (*P* value < 0.01)*.

### The Differences in Clinical Outcomes by Sex

We analyzed the clinical outcomes in male and female patients with septic shock (see Table 3). The in-hospital mortality rate was higher in women than in men (55.54 vs. 49.29%, *p* < 0.01). Meanwhile, male patients had higher hospital expenses (*p* < 0.01) and longer stays (*p* > 0.01) at the hospital.

**Table 3:** Sex-Based Differences in clinical outcomes of patients with septic shock.

**Clinical outcome**	**Total (*n* = 3,643)**	**Men (*n* = 2,345)**	**Women (*n* = 1,298)**	**Test value**	***p*** **value**
Death (*n*) and in-hospital mortality (%)	1,877 (51.52)	1,156 (49.29)	721(55.54)	13.070	0.000※
LOS [days, M (P25–P75)]	21.64 (6.00, 25.00)	22.71 (6.00, 25.00)	19.72 (6.00, 24.00)	−1.713	0.087
Expenses [rmb, M (P25–P75)]	100,064.21 (13,857.64, 123,022.83)	102,804.68 (33,063.69, 125,903.69)	95,113.20 (26,739.77, 118,980.72)	−4.274	0.000※

※ *means that the differences is statistically significant (*P* value < 0.01)*.

### Sex and In-hospital Mortality

We divided patients with septic shock into two groups according to different clinical outcomes (death or survival in the hospital) and compared the data of the two groups (see Table 4). We found that sex, age, length of stay, marital status, medical insurance status, chronic pulmonary disease, malignant tumor, ischemic heart disease, and urinary tract infection were significantly different between the two groups (*p* < 0.01).

**Table 4:** Risk factors for in-hospital mortality in patients with septic shock.

	**Total**	**Survival**	**Death**	**Test value**	***P*** **value**
**Gender** (*n*, %)	3,643	1,766	1,877	13.070	0.000※
Men (n, %)	2,345	1,189 (50.7%)	1,156 (49.3%)		
women (*n*, %)	1,298	577 (44.5%)	721 (55.5%)		
**Age** [years, M (P25–P75)]	77.00 (62.00, 85.00)	74.00 (64.00, 85.00)	79.00 (63.00, 84.00)	−9.268	0.000※
**Expense** [rmb, M (P25–P75)]	100,064.21 (13,857.64, 123,022.83)	102,804.68(33,063.69, 125,903.69)	95,113.20 (26,739.77, 118,980.72)	−2.321	0.020
**LOS** [days, M (P25-P75)]	21.64 (6.00, 25.00)	22.71 (6.00, 25.00)	19.72 (6.00, 24.00)	−5.167	0.000※
**Marital status**	3,643	1,766	1,877	35.404	0.000※
Unmarried (*n*, %)	228	154 (67.5%)	74 (32.5%)		
Married (*n*, %)	3,415	1,612 (47.2%)	1,803 (52.8%)		
**Race**	3,643	1,766	1,877	1.063	0.302
Han (*n*, %)	3,642	1,765 (48.5%)	1,877 (51.5%)		
Non-Han (*n*, %)	1	1 (100%)	0		
**Insurance**	3,643			29.585	0.000※
Medical insurance (n, %)	3,304	1,554 (47.0%)	1,750 (53.0%)		
Self-pay (*n*, %)	339	212 (62.5%)	127 (37.5%)		
**Level of hospital**				6.181	0.013
Second hospital (*n*, %)	399	170 (42.6%)	229 (57.4%)		
Tertiary Hospital (n, %)	3,244	1,596 (49.2%)	1,648 (50.8%)		
**Comorbidity**					
Hypertension (*n*, %)	1,803	852 (47.3%)	951 (52.7%)	2.134	0.144
Ischemic heart disease (*n*, %)	1,632	745 (45.6%)	887 (54.4%)	9.460	0.002※
DM (*n*, %)	1,351	672 (49.7%)	679 (50.3%)	1.374	0.241
CKD (*n*, %)	1,201	581 (48.4%)	620 (51.6%)	0.007	0.932
Malignant tumor (*n*, %)	707	291 (41.2%)	416 (58.8%)	18.803	0.000※
Liver disease (*n*, %)	1,347	664 (49.3%)	683 (50.7%)	0.573	0.449
Chronic pulmonary disease (*n*, %)	180	43 (23.9%)	137 (76.1%)	45.833	0.000※
**Site of infection**					
Pulmonary (*n*, %)	933	477 (51.1%)	456 (48.9%)	3.523	0.061
Skin (*n*, %)	74	36 (48.6%)	38 (51.4%)	0.001	0.976
Urinary tract (*n*, %)	802	467 (58.2%)	335 (41.8%)	39.165	0.000※
Gastrointestinal tract (*n*, %)	94	57 (60.6%)	37 (39.4%)	5.714	0.017
Abdominal cavity (*n*, %)	418	203 (48.6%)	215 (51.4%)	0.001	0.969
Blood stream (*n*, %)	74	36 (48.6%)	38 (51.4%)	0.001	0.976

※ *means that the differences is statistically significant (*P* value < 0.01)*.

We then performed univariate and multivariate logistic regression on the above different indicators (see Table 5). To examine the association between sex and in-hospital mortality, logistic regression models were used to adjust for patients' clinical characteristics, including sex, age, length of stay, marital status, medical insurance status, chronic pulmonary disease, malignant tumor, ischemic heart disease, and urinary tract infection.

**Table 5:** Logistics regression on the indicators of in-hospital death.

**Characteristic**	**Univariate analysis**			**Multivariate analysis**		
	**OR**	**95 %CI**	***p*** **value**	**OR**	**95 %CI**	***p*** **value**
Gender	0.778	(0.679, 0.892)	0.000※	0.732	(0.635, 0.844)	0.000※
Age	1.019	(1.016, 1.023)	0.000※	1.026	(1.021, 1.031)	0.000※
LOS	1.000	(0.999, 1.001)	0.395			
Marital status	0.430	(0.323, 0.571)	0.000※	1.663	(1.125, 2.457)	0.011
Insurance	1.880	(1.493, 2.367)	0.000※	1.390	(1.080, 1.789)	0.010
Ischemic heart disease	0.814	(0.714, 0.928)	0.002※	1.035	(0.887, 1.207)	0.663
Chronic pulmonary disease	0.317	(0.224, 0.449)	0.000※	0.349	(0.244, 0.499)	0.000※
Malignant tumor	0.693	(0.587, 0.818)	0.000※	0.640	(0.538, 0.761)	0.000※
Urinary tract infection	1.655	(1.412, 1.939)	0.000※	2.072	(1.745, 2.460)	0.000※

※ *means that the differences is statistically significant (*P* value < 0.01)*.

In univariate logistic regression, the B value of gender was −0.251, whereas in multivariate regression the B value of sex was −0.312. The results suggested that after adjusting the covariates, the correlation between gender and in-hospital death was greater. Male patients had a lower likelihood of hospital mortality than female patients (OR = 0.732, 95% CI = 0.635–0.844, *p* = 0.000).

## Discussion

In this large, hospital-based registry for male and female patients discharged with septic shock in Beijing China, we observed that men with septic shock were more likely to suffer from chronic diseases (such as hypertension, DM, ischemic heart disease, chronic kidney disease, chronic pulmonary disease, liver disease, malignant tumor) and had higher hospital expenses and longer stays at the hospital. However, the in-hospital mortality rate of male patients with septic shock was lower than that of female patients.

Previous animal and human studies indicated that females have advantageous immunologic and cardiovascular responses during infectious challenge, which means a higher sepsis incidence in males than in females. However, clinical studies on sex and mortality among critically ill sepsis patients have shown conflicting results (12). This may be related to the differences in study design, sample sizes, population included in the studies (ICU patients or non-ICU patients) and the research methods.

To explain the results in our study, we tried to analyze the following possible mechanisms. Estrogens have been proven to have a direct protective effect on vascular endothelial cells (13), inhibit endothelial cell apoptosis, induce endothelial cell proliferation and migration, and promote microvessel regeneration (14). However, estrogens also have physiologic actions that could be detrimental in sepsis (15). In studies of gender-specific responses to endotoxin, there were higher estrogen concentrations in elderly critically ill women than in younger critically ill women, as well as elevated estrogen concentrations in critically ill men, plus the association of higher estrogen levels with higher mortality in both women and men (16, 17). Non-biological explanations for our findings must also be considered. Previous studies (5, 18, 19) suggest that female patients with sepsis/septic shock received less medical care than male patients, and the proportion of withheld or withdrawn treatment was greater for female than for male patients. Although our study did not include data about treatment, it may also be one of the reasons why the in-hospital mortality of female patients with septic shock is higher than that of male patients (20). Sex differences in sites of infection were observed in the study, but similar to the hospital mortality difference, it is unclear whether they originate from gender differences in biology, comorbidity, or medical assessment and care.

The study was a large retrospective study of sample size, making the results credible. Many previous studies (18–21) indicated that the authors only had access to data for patients who presented with sepsis in the ED or ICU. However, in clinical practice, not all patients with septic shock receive treatment in the ED or ICU, so some cases may be missed. Our study included all patients with septic shock in all departments compared with other studies.

Limited by our current capabilities and the extent to which the database can be used, our research discovered a phenomenon but cannot fully explain its pathophysiological mechanism. Information on medical care during hospitalization cannot be fully indicated. Whether to adopt standardized treatment is very important for clinical prognosis. The results of this study can provide ideas and evidence for follow-up research. The patients in our study were middle-aged or elderly, which may not fully represent the characteristics of the entire adult population. This is a limitation of retrospective research. In our study, the death group was older than the alive group. As age increased, mortality also increased. We should consider the impact of age on mortality. However, there was no statistically significant difference in age between male and female patients. The impact of sex on in-hospital mortality was adjusted by logistic regression. In multivariate logistic regression, the B value of sex was greater than that in univariate regression. Based on this, we believe that the conclusions are valid.

## Conclusion

In this study, female patients with septic shock had a higher in-hospital mortality than male patients. This difference remained after multivariable adjustment.

## Data Availability Statement

The original contributions presented in the study are included in the article/Supplementary Material, further inquiries can be directed to the corresponding author.

## Ethics Statement

The studies involving human participants were reviewed and approved by Beijing Friendship Hospital Ethics Committee (No. 2021-P2-184-01). Written informed consent for participation was not required for this study in accordance with the national legislation and the institutional requirements.

## Author Contributions

MLD and XZ contributed to conception and design of the study. ZL, PL, and NZ organized the database. NZ and XZ performed the statistical analysis. XZ wrote the first draft of the manuscript. All authors contributed to manuscript revision, read, and approved the submitted version.

## Conflict of Interest

The authors declare that the research was conducted in the absence of any commercial or financial relationships that could be construed as a potential conflict of interest. The reviewer JL declared a shared affiliation with the authors to the handling editor at the time of the review.

## Publisher's Note

All claims expressed in this article are solely those of the authors and do not necessarily represent those of their affiliated organizations, or those of the publisher, the editors and the reviewers. Any product that may be evaluated in this article, or claim that may be made by its manufacturer, is not guaranteed or endorsed by the publisher.

## Abbreviations

LOS: length of hospital stay
ICU: Intensive care unit.
