# Supplementary material for: Sex Differences in In-hospital Mortality of Patients With Septic Shock: An Observational Study Based on Data Analysis From a Cover Sheet of Medical Records in Beijing
Source: Front Med (Lausanne). 2021 Oct 11;8:733410. doi: 10.3389/fmed.2021.733410 (PMC8542919; doi:10.3389/fmed.2021.733410)
Supplement: Supplementary file 1 [file Table_1.DOCX]

Table 1 Clinical characteristics of the septic shock patients at discharge

|  | **Total**  (n=3643) | **Men**  (n=2345) | **Women**  (n=1298) | **Test value** | ***p* value** |
| --- | --- | --- | --- | --- | --- |
| **Age**([years, M(P25-P75)]) | 77.00(62.00,85.00) | 78.00(62.00,85.00) | 77.00(63.00,84.00) | -1.257 | 0.209 |
| **Marital status** |  |  |  | 0.214 | 0.644 |
| Unmarried(n,%) | 228 | 150(65.8%) | 78(34.2%) |  |  |
| Married(n,%) | 3415 | 2195(64.3%) | 1220(35.7%) |  |  |
| **Race** |  |  |  | 1.807 | 0.179 |
| Han(n,%) | 3642 | 2345(64.4%) | 1297(35.6%) |  |  |
| Non-Han(n,%) | 1 | 0 | 1(100%) |  |  |
| **Insurance** |  |  |  | 3.283 | 0.070 |
| medical insurance(n,%) | 3304 | 2142(64.8%) | 1162(35.2%) |  |  |
| Self-pay(n,%) | 339 | 203(59.9%) | 136(40.1%) |  |  |
| **Level of hospital** |  |  |  | 0.958 | 0.328 |
| Second hospital(n,%) | 399 | 248(62.2%) | 151(37.8%) |  |  |
| Tertiary hospital(n,%) | 3244 | 2097(64.6%) | 1147(35.4%) |  |  |
| **Comorbidity** |  |  |  |  |  |
| Hypertension(n,%) | 1803 | 1174(65.1%) | 629(34.9%) | 0.861 | 0.354 |
| Ischemic heart disease(n,%) | 1632 | 1058(64.8%) | 574(35.2%) | 0.271 | 0.603 |
| DM(n,%) | 1351 | 872(64.5%) | 479(35.5%) | 0.029 | 0.866 |
| CKD(n,%) | 1201 | 809(67.4%) | 392(32.6%) | 6.986 | 0.008※ |
| Malignant Tumor(n,%) | 707 | 498(70.4%) | 209(29.6%) | 14.086 | 0.000※ |
| Liver disease(n,%) | 1347 | 917(68.1%) | 430(31.9%) | 12.807 | 0.000※ |
| Chronic pulmonary disease(n,%) | 180 | 120(66.7%) | 60(33.3%) | 0.435 | 0.509 |

Table 2 Site of infection in different genders

| **Site of infection** | **Total**  **(n=3643)** | **Men**  (n=2345) | **Women**  (n=1298) | **Test value** | ***p* value** |
| --- | --- | --- | --- | --- | --- |
| Pulmonary  (n,%) | 933 | 642(68.8%) | 291(31.2%) | 10.782 | 0.001※ |
| Skin  (n,%) | 74 | 46(62.2%) | 28(37.8%) | 0.161 | 0.689 |
| Urinary tract  (n,%) | 802 | 518(64.6%) | 284(35.4%) | 0.021 | 0.884 |
| Gastrointestinal tract  (n,%) | 94 | 61(64.9%) | 33(35.1%) | 0.012 | 0.914 |
| Abdominal cavity  (n,%) | 418 | 272(65.1%) | 146(34.9%) | 0.101 | 0.750 |
| Blood stream  (n,%) | 74 | 46(62.2%) | 28(37.8%) | 0.161 | 0.689 |

Table 3 Sex-Based Differences in clinical outcomes of patients with septic shock

| Clinical outcome | Total  (n=3643) | Men  (n=2345) | Women  (n=1298) | Test value | *p* value |
| --- | --- | --- | --- | --- | --- |
| Death(n) and in-hospital mortality(%) | 1877(51.52) | 1156(49.29) | 721(55.54) | 13.070 | 0.000※ |
| LOS[days, M(P25-P75)] | 21.64  (6.00,25.00) | 22.71  (6.00,25.00) | 19.72  (6.00,24.00) | -1.713 | 0.087 |
| Expenses[rmb, M(P25-P75)] | 100064.21  (13857.64,123022.83) | 102804.68  (33063.69,125903.69) | 95113.20  (26739.77,118980.72) | -4.274 | 0.000※ |

Table 4 Risk factors for in-hospital mortality in patients with septic shock

|  | **Total** | Survival | Death | **Test value** | **P value** |
| --- | --- | --- | --- | --- | --- |
| **Gender**(n,%) | 3643 | 1766 | 1877 | 13.070 | 0.000※ |
| Men(n,%) | 2345 | 1189(50.7%) | 1156(49.3%) |  |  |
| women(n,%) | 1298 | 577(44.5%) | 721(55.5%) |  |  |
| **Age**([years, M(P25-P75)]) | 77.00(62.00,85.00) | 74.00(64.00,85.00) | 79.00(63.00,84.00) | -9.268 | 0.000※ |
| **Expense**  [rmb, M(P25-P75)] | 100064.21  (13857.64,123022.83) | 102804.68  (33063.69,125903.69) | 95113.20  (26739.77,118980.72) | -2.321 | 0.020 |
| **LOS**  [days, M(P25-P75)] | 21.64(6.00,25.00) | 22.71(6.00,25.00) | 19.72(6.00,24.00) | -5.167 | 0.000※ |
| **Marital status** | 3643 | 1766 | 1877 | 35.404 | 0.000※ |
| unmarried(n,%) | 228 | 154(67.5%) | 74(32.5%) |  |  |
| married(n,%) | 3415 | 1612(47.2%) | 1803(52.8%) |  |  |
| **Race** | 3643 | 1766 | 1877 | 1.063 | 0.302 |
| Han(n,%) | 3642 | 1765(48.5%) | 1877(51.5%) |  |  |
| Non-Han(n,%) | 1 | 1(100%) | 0 |  |  |
| **Insurance** | 3643 |  |  | 29.585 | 0.000※ |
| medical insurance(n,%) | 3304 | 1554(47.0%) | 1750(53.0%) |  |  |
| Self-pay(n,%) | 339 | 212(62.5%) | 127(37.5%) |  |  |
| **Level of hospital** |  |  |  | 6.181 | 0.013 |
| Second hospital  (n,%) | 399 | 170(42.6%) | 229(57.4%) |  |  |
| Tertiary hospital  (n,%) | 3244 | 1596(49.2%) | 1648(50.8%) |  |  |
| **Comorbidity** |  |  |  |  |  |
| Hypertension  (n,%) | 1803 | 852(47.3%) | 951(52.7%) | 2.134 | 0.144 |
| Ischemic heart disease  (n,%) | 1632 | 745(45.6%) | 887(54.4%) | 9.460 | 0.002※ |
| DM(n,%) | 1351 | 672(49.7%) | 679(50.3%) | 1.374 | 0.241 |
| CKD(n,%) | 1201 | 581(48.4%) | 620(51.6%) | 0.007 | 0.932 |
| Malignant Tumor(n,%) | 707 | 291(41.2%) | 416(58.8%) | 18.803 | 0.000※ |
| Liver disease  (n,%) | 1347 | 664(49.3%) | 683(50.7%) | 0.573 | 0.449 |
| Chronic pulmonary disease(n,%) | 180 | 43(23.9%) | 137(76.1%) | 45.833 | 0.000※ |
| **Site of infection** |  |  |  |  |  |
| Pulmonary(n,%) | 933 | 477(51.1%) | 456(48.9%) | 3.523 | 0.061 |
| Skin(n,%) | 74 | 36(48.6%) | 38(51.4%) | 0.001 | 0.976 |
| Urinary tract(n,%) | 802 | 467(58.2%) | 335(41.8%) | 39.165 | 0.000※ |
| Gastrointestinal tract(n,%) | 94 | 57(60.6%) | 37(39.4%) | 5.714 | 0.017 |
| Abdominal cavity(n,%) | 418 | 203(48.6%) | 215(51.4%) | 0.001 | 0.969 |
| Blood stream(n,%) | 74 | 36(48.6%) | 38(51.4%) | 0.001 | 0.976 |

Table 5 Logistics regression on the indicators of in-hospital death

| **Characteristic** | **Univariate analysis** | | | **Multivariate analysis** | | |
| --- | --- | --- | --- | --- | --- | --- |
|  | OR | 95%CI | *p* value | OR | 95%CI | *p* value |
| **Gender** | 0.778 | (0.679,0.892) | 0.000※ | 0.732 | (0.635,0.844) | 0.000※ |
| **Age** | 1.019 | (1.016,1.023) | 0.000※ | 1.026 | (1.021,1.031) | 0.000※ |
| **LOS** | 1.000 | (0.999,1.001) | 0.395 |  |  |  |
| **Marital status** | 0.430 | (0.323,0.571) | 0.000※ | 1.663 | (1.125,2.457) | 0.011 |
| **Insurance** | 1.880 | (1.493,2.367) | 0.000※ | 1.390 | (1.080,1.789) | 0.010 |
| **Ischemic heart disease** | 0.814 | (0.714,0.928) | 0.002※ | 1.035 | (0.887,1.207) | 0.663 |
| **Chronic pulmonary disease** | 0.317 | (0.224,0.449) | 0.000※ | 0.349 | (0.244,0.499) | 0.000※ |
| **Malignant tumor** | 0.693 | (0.587,0.818) | 0.000※ | 0.640 | (0.538,0.761) | 0.000※ |
| **Urinary tract infection** | 1.655 | (1.412,1.939) | 0.000※ | 2.072 | (1.745,2.460) | 0.000※ |
